# Comparison of Next-Generation Sequencing and Polymerase Chain Reaction for Personalized Treatment-Related Genomic Status in Patients with Metastatic Colorectal Cancer

**DOI:** 10.3390/cimb44040106

**Published:** 2022-04-05

**Authors:** Wei-Chih Su, Yi-Chen Tsai, Hsiang-Lin Tsai, Tsung-Kun Chang, Tzu-Chieh Yin, Ching-Wen Huang, Yen-Cheng Chen, Ching-Chun Li, Po-Jung Chen, Yun-Ru Liu, Tsung-Han Hsieh, Jaw-Yuan Wang

**Affiliations:** 1 Division of Colorectal Surgery, Department of Surgery, Kaohsiung Medical University Hospital, Kaohsiung Medical University, Kaohsiung 80756, Taiwan; lake0126@yahoo.com.tw (W.-C.S.); alicetsai88@gmail.com (Y.-C.T.); chunpin870132@yahoo.com.tw (H.-L.T.); tsungkunchang@gmail.com (T.-K.C.); ajaxyin@gmail.com (T.-C.Y.); baseball5824@yahoo.com.tw (C.-W.H.); googoogi05@gmail.com (Y.-C.C.); dobird05@yahoo.com.tw (C.-C.L.); glaudiotennis@gmail.com (P.-J.C.); 2 Graduate Institute of Clinical Medicine, College of Medicine, Kaohsiung Medical University, Kaohsiung 80756, Taiwan; 3 Department of Surgery, Faculty of Medicine, College of Medicine, Kaohsiung Medical University, Kaohsiung 80756, Taiwan; 4 Department of Surgery, Kaohsiung Municipal Tatung Hospital, Kaohsiung Medical University, Kaohsiung 80756, Taiwan; 5 Division of General and Digestive Surgery, Department of Surgery, Kaohsiung Medical University Hospital, Kaohsiung Medical University, Kaohsiung 80756, Taiwan; 6 Joint Biobank, Office of Human Research, Taipei Medical University, Taipei 10675, Taiwan; d90444002@tmu.edu.tw (Y.-R.L.); thhsieh@tmu.edu.tw (T.-H.H.); 7 Graduate Institute of Medicine, College of Medicine, Kaohsiung Medical University, Kaohsiung 80756, Taiwan; 8 Center for Cancer Research, Kaohsiung Medical University, Kaohsiung 80756, Taiwan; 9 Cohort Research Center, Kaohsiung Medical University, Kaohsiung 80756, Taiwan; 10 Pingtung Hospital, Ministry of Health and Welfare, Pingtung 90054, Taiwan; 11 Clinical Pharmacogenomics and Pharmacoproteomics, School of Pharmacy, Taipei Medical University, Taipei 11031, Taiwan

**Keywords:** next-generation sequencing, polymerase chain reaction, metastatic colorectal cancer

## Abstract

Personalized treatments based on the genetic profiles of tumors can simultaneously optimize efficacy and minimize toxicity, which is beneficial for improving patient outcomes. This study aimed to integrate gene alterations associated with predictive and prognostic outcomes in patients with metastatic colorectal cancer (mCRC) with polymerase chain reaction (PCR) and in-house next-generation sequencing (NGS) to detect *KRAS*, *NRAS*, and *BRAF* mutations. In the present study, 41 patients with mCRC were assessed between August 2017 and June 2019 at a single institution. The overall concordance between NGS and PCR results for detecting *KRAS*, *NRAS,* and *BRAF* mutations was considerably high (87.8–92.7%), with only 15 discrepant results between PCR and NGS. Our companion diagnostic test analyzes *KRAS*, *NRAS,* and *BRAF* as a panel of CRC molecular targets; therefore, it has the advantages of requiring fewer specimens and being more time and cost efficient than conventional testing for separate analyses, allowing for the simultaneous analysis of multiple genes.

## 1. Introduction

With approximately 1.93 million newly diagnosed cases and 935,000 deaths occurring in 2020, colorectal cancer (CRC) is the third most diagnosed cancer and the second leading cause of cancer death worldwide [1]. In Taiwan, CRC is among the most frequently diagnosed malignancies and the third leading cause of cancer death [2]. Fluorouracil (5-FU) administration is the established standard of care for patients with CRC, but the treatment landscape for metastatic cancer has evolved quickly after the approval of several targeted therapies, leading to improved tumor response rates and patient survival [3].

Molecular testing has provided new insights and guidance regarding oncological management in CRC due to the increased availability of new targeted therapies [4]. Therefore, such tests are routinely incorporated into CRC resection specimen reports [4]. Treatments for human cancer are increasingly incorporating precision medicine. Targeting the genomic status of the tumors with molecularly targeted therapies has become a common modality [5]. Drugs that target molecular pathways are available for patients who have CRC and exhibit relevant gene variations.

The introduction and approval of targeted therapies for metastatic CRC (mCRC) have led to improved patient outcomes [6]. Although various treatments are available, the outcomes and toxicity associated with each regimen can vary from patient to patient [7]. Personalized treatments based on genetic profiles of tumors can simultaneously optimize efficacy and minimize toxicity, which improves CRC treatment outcomes [6]. 

Established data from clinical trials have demonstrated that rat sarcoma virus (*RAS*) mutations in codons 12 and 13 predict a poor response to monoclonal antibodies (panitumumab/cetuximab) that target the epidermal growth factor receptors (*EGFR*s), which is detrimental to therapies that combine monoclonal antibodies with oxaliplatin- or irinotecan-based chemotherapy [6,8,9,10,11,12,13,14]. Numerous studies have shown that the proto-oncogene B-Raf (*BRAF*) mutation is associated with considerably poor clinical outcomes in patients with mCRC [15,16,17], regardless of the treatment modality that was adopted [18].

If a traditional analysis method such as polymerase chain reaction (PCR) is used to detect all the aforementioned molecular alterations, a larger amount of specimens is required, and the sample quality is likely to be low or variable; however, these drawbacks do not apply to next-generation sequencing (NGS) [5]. For example, NGS has advantages in the screening of targetable fusions that may trigger genomic events in 8–20% of advanced melanomas that lack distinctive driver mutations [19]. Similarly, NGS germline multigene panels including breast cancer gene 1 (*BRCA1*) and breast cancer gene 2 (*BRCA2*) genes can identify driver mutations and molecular targets to allow a personalized treatment of prostate cancer [20].

The present study aimed to compare PCR and in-house NGS companion diagnostic test results for gene alterations associated with predictive and prognostic outcomes in patients with mCRC.

## 2. Materials and Methods

### 2.1. Patients

**Figure 1:**
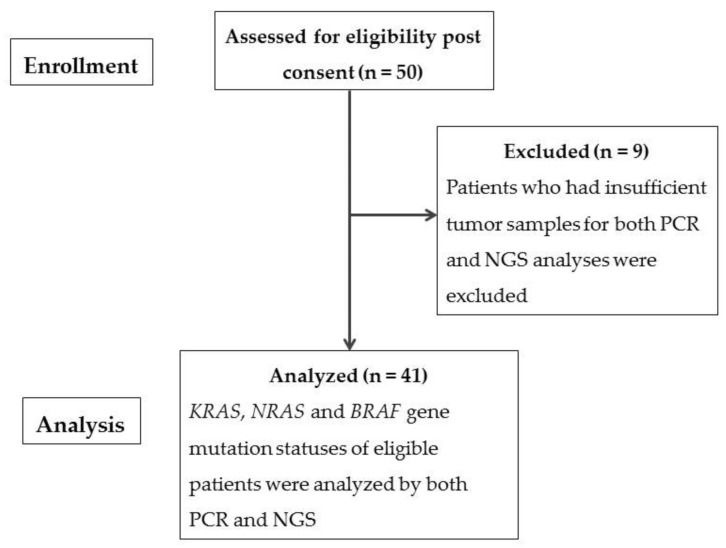
The Consolidated Standards of Reporting Trials (CONSORT) diagram of the present study.

In the present study, patients with mCRC were assessed between August 2017 and June 2019 at a single institution, and 50 patients were enrolled initially. The final sample comprised 41 patients with mCRC, after 9 patients who had insufficient tumor samples for both PCR and NGS analyses were excluded. The Consolidated Standards of Reporting Trials (CONSORT) diagram is shown in Figure 1.

Tumor, node, and metastasis (TNM) staging was performed per the eighth edition of the Cancer Staging Manual of the American Joint Commission on Cancer and the Union for International Cancer Control [21]. The patient data that were collected comprised age, sex, tumor location, TNM classification, presence of lymphovascular invasion, and presence of perineural invasion. 

The genetic profiles of the 41 patients (including their *KRAS*, *NRAS*, and *BRAF*) were accessed before a suitable regimen was selected for each patient. Patients with the *RAS* wild-type status were prescribed first-line regimens that were reimbursed by Taiwan’s government; the regimens were selected after discussions between the physician and patients, regarding available medical insurance reimbursement schemes. The regimens were as follows: (1) irinotecan plus fluorouracil/leucovorin (FOLFIRI) with bevacizumab, (2) FOLFIRI with cetuximab, and (3) FOLFOX with panitumumab. For patients with *RAS* mutations, FOLFIRI with bevacizumab was used as the first-line regimen. 

The evaluation of tumor response was performed per the Response Evaluation Criteria in Solid Tumors Version 1.1 guidelines [22,23] and typically conducted after every sixth cycle. When a *BRAF* mutation was detected, the treatment response was evaluated after the first four to six cycles of first-line therapy; when patients exhibited poor treatment response, their regimens were switched. Radiotherapy was administered to patients with a clinical diagnosis of mCRC with cT3–cT4, and/or clinical nodal stage N1–N2 rectal cancer if they were eligible for the treatment.

### 2.2. Ethics Approval

The present study was approved by the Ethical and Research Committee of Kaohsiung Medical University Hospital (approval no.: KMUHIRB-G(I)-20180049; obtained on 17 May 2019). The protocol was conducted per the Good Clinical Practice guidelines and institutional review board regulations that conformed to the 1983 revision of the Helsinki Declaration of 1975. 

### 2.3. Analyses of KRAS, NRAS, BRAF 

DNA was extracted by the QIAamp DNA Mini Kit (Qiagen, Hilden, Germany) according to the manufacturer’s instructions. All mutations are detected by using Sanger sequencing. The program for the PCR amplification in *KRAS*, *NRAS,* and *BRAF* involves 5 min of initial denaturation at 94 °C, 40 cycles of amplification consisting of 30 s at 94 °C, 30 s at 58 °C, and 30 s at 72 °C, with a final additional elongation at 72 °C for 7 min. PCR was performed using a LightCycler 480 (Roche Diagnostics, Wien, Austria). After amplification, the products were purified and directly sequenced by using the BigDye Terminator V.3.1 chemistries (Applied Biosystems, Foster City, CA, USA). Sequences were run on an ABI3130XL automated sequencer (Applied Biosystems). The primers used are listed in Supplementary Table S1. 

### 2.4. Sample Preparation for Genotyping of KRAS, NRAS, and BRAF Hotspot Mutations through In-House NGS

Tumor samples were obtained from the tissue sections of optimal cutting temperature compound (OCT compound)-embedded frozen tissue samples. DNA was extracted using a QI-Aamp DNA Mini Kit (Qiagen, Hilden, Germany). DNA quantification was performed using a Qubit dsDNA HS Assay Kit (Invitrogen, Waltham, MA, USA).

### 2.5. Detection of KRAS, NRAS, and BRAF Hotspot Mutations through In-House NGS

The detection of hotspot mutations was performed using an AmpliSeq Assay Kit. DNA was amplified using customized primers (IDT) covering hotspot mutations for *KRAS* codons 12, 13, 61, and 146; *NRAS* codons 12, 13, 59, 61, 117, and 146; and *BRAF* Val600Glu. The libraries were amplified using a KAPA HiFi HotStart ReadyMixPCR Kit (Roche) and Nextera XT Index Primers (Illumina, San Diego, CA, USA), and subsequently purified using Agencourt AMPure XP (Beckman). Sequencing was performed using a MiSeq System and MiSeq Reagent Kit v3 (Illumina). All library preparations were performed at the translational core facility of Taipei Medical University. After sequencing was completed, read files (fastq) were mapped to the Hg19 reference using BWA [24]. The BAM files of all samples were sorted and then converted to the mpileup file format by using SAMtools [25]. Subsequently, the hotspot mutations were called using Varscan (v2.4.3) [26] and the following criteria were applied: minimal coverage > 200× and minimal variant frequency > 5%. The amplicon sequence of the mutational hotspots in *KRAS*, *NRAS,* and *BRAF* are listed in Supplementary Table S2. The figures that display the NGS results were visualized using Integrative Genomic Viewer (IGV) (version 2.12.2; © 2013–2021 Broad Institute and the Regents of the University of California).

### 2.6. Statistical Analysis

Analyses were performed using Microsoft Excel 2010. Continuous variables were ex-pressed as medians and interquartile ranges (IQRs) and analyzed using a one-way analysis of variance. 

## 3. Results

### 3.1. Patient Demographics 

The median (IQR) age of the 41 patients with mCRC in the present study was 65 (34−88) years and their male–female ratio was 1.73. The left side of the colon (defined as the area from the splenic flexure to the sigmoid colon) was the most common tumor site (85.3%). Vascular and perineural invasions were present in 10 (24.4%) and 12 (29.3%) patients, respectively (Table 1).

#### Comparison of PCR and NGS for Testing of Targeted Genes

Table 2 presents a comparison of the PCR and NGS genetic analysis results obtained from 41 patients with mCRC. The Kirsten rat sarcoma virus (*KRAS*), neuroblastoma RAS viral oncogene homolog (*NRAS*), and *BRAF* genes were subjected to both PCR and NGS tests. The discrepancies between the PCR and NGS results (Table 2) are presented in Supplementary Figures S1–S15. Table 3 reveals the high concordance (87.8–92.7%) between the detection results (*KRAS*, *NRAS*, and *BRAF* mutations) obtained through NGS and those obtained through PCR. Among the 41 patients with mCRC, three *KRAS* mutations were detected by PCR but not by NGS (Table 3). Similarly, among 39 patients with mCRC who were tested for *NRAS* mutations, three *NRAS* mutations were detected by NGS but not by PCR (Table 3). *BRAF* mutations in five patients were detected by NGS but not by PCR (Table 3). Table 4 summarizes the *KRAS*, *NRAS,* and *BRAF* mutations detected by both PCR and NGS in the present study. Examples of all gene mutations presented in Table 4 are provided in Supplementary Figures S16–S21.

## 4. Discussion

We compared PCR and in-house NGS test results for *KRAS*, *NRAS,* and *BRAF* mutation detection in patients with mCRC. Overall, the concordance between the PCR and in-house NGS results was high. Only 15 discrepant results were detected between PCR and in-house NGS (Table 2). Three variants of the *KRAS* gene were detected only by PCR (Table 3). Three variants of the *NRAS* gene and five variants of the *BRAF* gene were detected only by NGS (Table 3). This suggests that NGS is more sensitive to detecting genetic mutations than real-time PCR is. 

Multistep processes associated with histological, morphological, and genetic modifications that emerge with age contribute to the development of CRC [27]. Activated RAS–RAF–MAPK and PI3K–PTEN–AKT signaling pathways play a crucial role in modulating cell proliferation, angiogenesis, cell motility, and apoptosis [28,29]. The cumulative mutations in tumor suppressor genes and proto-oncogenes (including *KRAS*, *NRAS*, *BRAF*, and *PIK3CA*) in RAS–RAF–MAPK and PI3K–PTEN–AKT signaling pathways lead to the development of CRC [30,31]. 

Cetuximab and panitumumab, which are monoclonal antibodies against *EGFR*, have been used since 2004 to treat mCRC [32]. *KRAS* mutation is present in nearly 30–45% of CRC tumors, and mutant *KRAS* is associated with resistance to anti-*EGFR* therapies [33,34,35,36]. *KRAS* codons 12 and 13 are the two most common hotspots, accounting for approximately 95% of all *KRAS* mutation types (approximately 80% and 15% of such mutations occur in codons 12 and 13, respectively) [34,35]. Patients with *KRAS*-mutated metastatic colorectal tumors are resistant to cetuximab and have shorter progression-free survival and overall survival durations when compared with patients without such mutations [37,38,39]. 

The downstream effectors of the *EGFR* signaling pathway include *BRAF* and *NRAS*, and the components of the *PI3K* signaling pathway can trigger negative responses to anti-EGFR treatments [33,40]. Notably, right-sided CRC has a significantly poorer prognosis relative to left-sided CRC, and it is often unresponsive to *EGFR* inhibition [41]. Compared with left-sided mCRC, right-sided mCRC with microsatellite stability is more likely to present either a hotspot *RAS* mutation or *BRAF* V600E (80% for right-sided mRC vs. 46% for left-sided mCRC [41,42]. Conversely, mutations in Adenomatous polyposis coli (*APC*) and tumor protein p53 (*TP53*), as well as amplifications of Erb-B2 Receptor Tyrosine Kinase 2 (*ERBB2*) and *EGFR*, are more frequently observed in left-sided CRC [43]. The expressions of *EGFR* ligands epiregulin (*EREG*) and amphiregulin (*AREG*) are also significantly higher in left-sided CRC [43].

Another member of the *RAS* family is *NRAS*, which has effector-binding domains that are identical to those in *KRAS* and yield similar mutational effects [44,45]. The Panitumumab Randomized Trial in Combination with Chemotherapy for Metastatic Colorectal Cancer to Determine Efficacy (PRIME) trial reported the detrimental effect of adding panitumumab to first-line FOLFOX in patients with *RAS* mutations [10]. Patients with *NRAS* mutations respond poorly and are resistant to anti-*EGFR* monoclonal antibody therapy [16]. 

Studies have shown that the *BRAF* mutation is associated with aggressive tumors and poor prognosis in patients with mCRC [15,16,17,18]. *BRAF* mutations are present almost exclusively in wild-type *KRAS* CRC, and they are present in 8.1% of patients with mCRC [36,46]. Among the eight mCRC patients with the *BRAF* codon 600 mutation in the present study, all except one patient presented *KRAS* wild-type results. The V600E mutation of *BRAF* represents a significant, independent, and negative prognostic factor for mCRC [36,47].

*BRAF* may be a negative prognostic factor for patients who have CRC and have received hepatic or pulmonary metastasectomy [48]. Poor efficacy of *BRAF* inhibitors in monotherapy for patients with mCRC and *BRAF* mutation was reported [49]. Therefore, detecting the *BRAF* mutation can help to predict poor treatment response. Evaluations of treatment response in patients with *BRAF*-mutated CRC are conducted after the first four to six cycles of first-line therapy. When a poor response is noted in patients with *BRAF*-mutated CRC, a second-line regimen is implemented.

In the present study, the genetic profiles of patients with mCRC aided the personalization of regimens to enhance treatment efficacy and minimize treatment toxicity, which led to improved overall and disease-free survival among patients with CRC. Based on our experience in the clinical setting, initial treatment considerations for patients with mCRC are generally determined by first testing the *RAS* gene status of such patients [36]. We published results in February 2022 on the personalized regimens of patients with wild-type *RAS* mCRC, and we reported that they could tolerate escalated doses of irinotecan based on the results of *UGT1A1* testing and potentially achieve a more favorable clinical outcome without a significantly increased exposure to toxicity [50].

The NGS-based companion diagnostic test conducted in the present study analyzed *KRAS*, *NRAS*, and *BRAF* together as a panel of CRC molecular targets; therefore, it has the advantages of requiring fewer specimens and being more time and cost efficient in determining the optimal regimen for each patient. Implementing individualized treatment plans for patients with mCRC on the basis of their molecular testing results is essential for achieving optimal clinical outcomes.

The discrepancies between PCR and NGS results may be due to the low tumor content of the specimens and prolonged nucleic acid conversion, which may have led to false negative PCR results. The false negative rates of NGS in the discrepant results may have been influenced by pipeline parameters and read coverage [51]. Discrepancies can be confirmed using Sanger sequencing, but this was not performed in the present study and represents one of the study’s limitations. However, the discrepant results were generated from multiple mutation sites, indicating the absence of bias for any mutation.

## 5. Conclusions

Our results suggest that NGS, which produced results that shared high concordance with those produced by PCR, can be used to investigate the status of popular druggable genes associated with CRC and be expanded to include more genes. Given the limited sample size and data (collected from a single institution) used in the present study, future studies could conduct similar research using larger sample sizes.

## Figures and Tables

**Table 1:** Demographics of study patients (*n* = 41).

	Subjects with Metastatic CRC (*n* = 41)
Median age (years, range)	65 (34–88)
Male	64 (34–86)
Female	65 (38–88)
Gender	
Male	26 (63.4%)
Female	15 (36.6%)
Tumor location	
R’t colon/L’t colon	6 (14.6%)/35 (85.3%)
Depth of tumor invasion	
T1/T2/T3/T4	0/2 (4.9%)/27 (65.8%)/12 (29.3%)
Lymph node involvement	
N0/N1/N2	11 (26.8%)/17 (41.5%)/13 (31.7%)
Vascular invasion	
Yes/No	10 (24.4%)/31 (75.6%)
Perineural invasion	
Yes/No	12 (29.3%)/29 (70.7%)

CRC, Colorectal cancer; R’t colon, right-sided colon was defined as the region from the cecum to the splenic flexure; L’t colon, left-sided colon was defined as the region from the splenic flexure to the rectum.

**Table 2:** Comparison of PCR versus NGS for testing of targeted genes in patients with mCRC (*n* = 41).

		Somatic Mutation					
Case No.	Tumor Content (%)	*KRAS* Condon 12/13/59/61/146 Tested by PCR	*KRAS* Condon 12/13/61/146 Tested by NGS	*NRAS* Condon 12/13 Tested by PCR	*NRAS* Condon 12/13 Tested by NGS	*BRAF* Codon 600 Tested by PCR	*BRAF* Codon 600 Tested by NGS
**1**	30	wild-type	wild-type	wild-type	wild-type	wild-type	wild-type
**2**	**<5**	**codon 61 mutation**	**wild-type**	wild-type	wild-type	wild-type	wild-type
**3**	90	codon 12 mutation	codon 12 mutation	no data *	wild-type	wild-type	wild-type
**4**	15	wild-type	wild-type	wild-type	wild-type	wild-type	wild-type
**5**	70	wild-type	wild-type	wild-type	wild-type	wild-type	wild-type
**6**	50	wild-type	wild-type	wild-type	wild-type	wild-type	wild-type
**7**	10	**wild-type**	**codon 61 mutation**	wild-type	wild-type	wild-type	wild-type
**8**	20	codon 12 mutation	codon 12 mutation	no data *	codon 12 mutation	wild-type	wild-type
**9**	30	wild-type	wild-type	wild-type	wild-type	wild-type	wild-type
**10**	<5	wild-type	wild-type	wild-type	wild-type	wild-type	wild-type
**11**	<5	wild-type	wild-type	wild-type	wild-type	wild-type	wild-type
**12**	20	wild-type	wild-type	wild-type	wild-type	**codon 597 mutation**	**codon 600 mutation**
**13**	70	**codon 59 mutation**	**codon 13 mutation**	wild-type	wild-type	**wild-type**	**codon 600 mutation**
**14**	40	codon 146 mutation	codon 146 mutation	wild-type	wild-type	wild-type	wild-type
**15**	<5	wild-type	wild-type	wild-type	wild-type	wild-type	wild-type
**16**	10	wild-type	wild-type	**wild-type**	**codon 12 mutation**	**wild-type**	**codon 600 mutation**
**17**	60	wild-type	wild-type	**wild-type**	**codon 12 mutation**	**wild-type**	**codon 600 mutation**
**18**	20	codon 61 mutation	codon 61 mutation	wild-type	wild-type	wild-type	wild-type
**19**	60	wild-type	wild-type	**wild-type**	**codon 12 mutation**	codon 600 mutation	codon 600 mutation
**20**	30	wild-type	wild-type	codon 12 mutation	codon 12 mutation	**wild-type**	**codon 600 mutation**
**21**	15	wild-type	wild-type	wild-type	wild-type	codon 600 mutation	codon 600 mutation
**22**	10	wild-type	wild-type	wild-type	wild-type	**wild-type**	**codon 600 mutation**
**23**	10	codon 12 mutation	codon 12 mutation	wild-type	wild-type	wild-type	wild-type
**24**	40	wild-type	wild-type	wild-type	wild-type	wild-type	wild-type
**25**	10	wild-type	wild-type	wild-type	wild-type	wild-type	wild-type
**26**	30	codon 12 mutation	codon 12 mutation	wild-type	wild-type	wild-type	wild-type
**27**	20	wild-type	wild-type	wild-type	wild-type	wild-type	wild-type
**28**	40	wild-type	wild-type	wild-type	wild-type	wild-type	wild-type
**29**	80	wild-type	wild-type	wild-type	wild-type	wild-type	wild-type
**30**	10	**codon 13 mutation**	**wild-type**	wild-type	wild-type	wild-type	wild-type
**31**	<5	**codon 12 mutation**	**wild-type**	wild-type	wild-type	wild-type	wild-type
**32**	95	codon 13 mutation	codon 13 mutation	wild-type	wild-type	wild-type	wild-type
**33**	<5	wild-type	wild-type	wild-type	wild-type	wild-type	wild-type
**34**	<5	wild-type	wild-type	wild-type	wild-type	wild-type	wild-type
**35**	20	**codon 61 mutation**	**wild-type**	wild-type	wild-type	wild-type	wild-type
**36**	50	wild-type	wild-type	wild-type	wild-type	wild-type	wild-type
**37**	80	codon 146 mutation	codon 146 mutation	wild-type	wild-type	wild-type	wild-type
**38**	30	wild-type	wild-type	wild-type	wild-type	wild-type	wild-type
**39**	30	wild-type	wild-type	wild-type	wild-type	wild-type	wild-type
**40**	95	codon 12 mutation	codon 12 mutation	wild-type	wild-type	wild-type	wild-type
**41**	40	wild-type	wild-type	wild-type	wild-type	wild-type	wild-type

Bolded terms indicate inconsistencies in results between PCR and NGS results. * Two cases had no PCR data on *NRAS.*

**Table 3:** Detection of *KRAS*, *NRAS*, and *BRAF* mutation by PCR versus NGS in patients with mCRC.

Gene	No. of Cases Compared	Wild-Type Detected by PCR	Wild-Type Detected by NGS	Mutation Detected by PCR	Mutation Detected by NGS	Percentage of Concordance (%)
*KRAS*	41	27	30	14	11	92.7
NRAS *	39	38	35	1	4	92.3
*BRAF*	41	38	33	3	8	87.8

* Two cases had no PCR data on *NRAS.*

**Table 4:** *KRAS*, *NRAS*, and *BRAF* mutations detected by PCR and NGS in patients with mCRC (*n* = 41).

			No. of Patients with mCRC	
Gene	Source	Mutation	Real-Time PCR	NGS
*KRAS*	Tissue DNA	Codon 12	6 (14.6%)	5 (12.2%)
		Codon 13	2 (4.9%)	2 (4.9%)
		Codon 59	1 (2.4%)	0
		Codon 61	3 (7.3%)	2 (4.9%)
		Codon 146	2 (4.9%)	2 (4.9%)
**Total**			14 (34.1%)	11 (26.8%)
*NRAS*	Tissue DNA	Codon 12	1 (2.4%)	5 (12.2%)
**Total**			1 (2.4%)	5 (12.2%)
*BRAF*	Tissue DNA	Codon 600 Codon 597	2 (4.9%) 1 (2.4%)	8 (19.5%) 0
**Total**			3 (7.3%)	8 (19.5%)

## Data Availability

The data presented in this study are available on request from the corresponding author.

## Supplementary Materials

The following supporting information can be downloaded at: https://www.mdpi.com/article/10.3390/cimb44040106/s1, Figures S1–S21: Experimental data on decrepencies are displayed in Figures S1–S15; Example of various gene mutations detected by NGS visualized by Integrative Genomic Viewer (IGV) are displayed in Figures S16–S20; Electropherogram examples of various gene mutations are displayed in Figure S21. Table S1: The primers used in PCR amplification of KRAS, NRAS, and BRAF, Table S2: Amplicon sequence of mutational hotspots in KRAS, NRAS, and BRAF.
