# Supplementary material for: Comparison of Next-Generation Sequencing and Polymerase Chain Reaction for Personalized Treatment-Related Genomic Status in Patients with Metastatic Colorectal Cancer
Source: Curr Issues Mol Biol. 2022 Apr 5;44(4):1552–63. doi: 10.3390/cimb44040106 (PMC9164059; doi:10.3390/cimb44040106)
Supplement: Supplementary file 1 [file cimb-44-00106-s001.zip › 5. Supplementary figure legends-20220314.pdf]

Supplementary figure legends

Supplementary figure S1. *KRAS* status of case no. 2 detected by both PCR and NGS.

Supplementary figure S2. *KRAS* status of case no. 7 detected by both PCR and NGS.

Supplementary figure S3. *BRAF* status of case no. 12 detected by both PCR and NGS.

Supplementary figure S4. *KRAS* status of case no. 13 detected by both PCR and NGS.

Supplementary figure S5. *BRAF* status of case no. 13 detected by both PCR and NGS.

Supplementary figure S6. *NRAS* status of case no. 16 detected by both PCR and NGS.

Supplementary figure S7. *BRAF* status of case no. 16 detected by both PCR and NGS.

Supplementary figure S8. *NRAS* status of case no. 17 detected by both PCR and NGS.

Supplementary figure S9. *BRAF* status of case no. 17 detected by both PCR and NGS.

Supplementary figure S10. *NRAS* status of case no. 19 detected by both PCR and NGS.

Supplementary figure S11. *BRAF* status of case no. 20 detected by both PCR and NGS.

Supplementary figure S12. *BRAF* status of case no. 22 detected by both PCR and NGS.

Supplementary figure S13. *KRAS* status of case no. 30 detected by both PCR and NGS.

Supplementary figure S14. *KRAS* status of case no. 31 detected by both PCR and NGS.

Supplementary figure S15. *KRAS* status of case no. 35 detected by both PCR and NGS.

Supplementary figure S16. An example of *KRAS* gene mutation in codon 12 (case no. 3) detected by NGS visualized by Integrative Genomic Viewer (IGV).

Supplementary figure S17. An example of *KRAS* gene mutation in codon 13 (case no. 32) detected by NGS visualized by Integrative Genomic Viewer (IGV).

Supplementary figure S18. An example of *KRAS* gene mutation in codon 61 (case no. 7) detected by NGS visualized by Integrative Genomic Viewer (IGV).

Supplementary figure S19. An example of *KRAS* gene mutation in codon 146 (case no. 14) detected by NGS visualized by Integrative Genomic Viewer (IGV).

Supplementary figure S20.

(a) An example of *NRAS* gene mutation in codon 12 (case no. 17) detected by NGS visualized by Integrative Genomic Viewer (IGV).

(b) An example of *BRAF* gene mutation in codon 600 detected by NGS visualized by Integrative Genomic Viewer (IGV).

Supplementary figure S21.

(a) An electropherogram example of *KRAS* gene mutation in codon 12 (case no. 31).

(b) An electropherogram example of *KRAS* gene mutation in codon 13 (case no. 30).

(c) An electropherogram example of *KRAS* gene mutation in codon 59 (case no. 13).

(d) An electropherogram example of *KRAS* gene mutation in codon 61 (case

no. 37).

(e) An electropherogram example of *KRAS* gene mutation in codon 146 (case no. 14).

(f) An electropherogram example of *NRAS* gene mutation in codon 12 (case no. 20).

(g) An electropherogram example of *BRAF* gene mutation in codon 600 (case no. 19).

(h) An electropherogram example of *BRAF* gene mutation in codon 597 (case no. 12).
