# Supplementary material for: Comparison of Next-Generation Sequencing and Polymerase Chain Reaction for Personalized Treatment-Related Genomic Status in Patients with Metastatic Colorectal Cancer
Source: Curr Issues Mol Biol. 2022 Apr 5;44(4):1552–63. doi: 10.3390/cimb44040106 (PMC9164059; doi:10.3390/cimb44040106)
Supplement: Supplementary file 1 [file cimb-44-00106-s001.zip › Supplementary table 2.pdf]

Supplementary Table S2. Amplicon sequence of mutational hotspots in *KRAS*, *NRAS*, and *BRAF*.

Target genes	Mutational hotspots	Hg19	Amplicon sequence
KRAS	Exon 2 (codon12, 13)	chr12:25398284, chr12:25398281	CAAAGAATGGTCCTGCACCAGTAATATG CATATTAAAACAAGATTTACCTCTATTGTT GGATCATATTCGTCCACAAAATGATTCTG AATTAGCTGTATCGTCAAGGCACTCTTGC CTACGCCACCAGCTCCAACTACCACAAG TTTATATTCAGTCATTTTCAGCAGGCCT
	Exon 3 codon 61	chr12:25380276	TCCTCATGTACTGGTCCCTCATTGCACTGT ACTCCTCTTGACCTGCTGTGTCGAGAATA TCCAAGAGACAGGTTTCTCCATCAATTAC TACTTGCTTCCTGTAGGAATCCTGAGAAG GGAGAAACACAGTCTGGATTATTACAGT GCACCTTTTAC
	Exon 4 codon 146	chr12:25378561	CAGATCTGTATTTATTTCAGTGTTACTTAC CTGTCTTGTCTTTGCTGATGTTTCAATAAA AGGAATTCCATAACTTCTTGCTAAGTCCT GAGCCTGTTTTGTGTCTACTGTTCTAGAA GGCAAATCACATTTATTTCCTACTAGGAC CATAGGTACATCTTCAGAGTC
NRAS	Exon 2 (codon 12, 13)	chr1:115258747, chr1:115258744	CCTCACCTCTATGGTGGGATCATATTCATC TACAAAGTGGTTCTGGATTAGCTGGATTG TCAGTGCGCTTTTCCCAACACCACCTGCT CCAACCACCACCAGTTTGTACTCAGTCAT TTCACACCAGCAAGAAC
	Exon 3 (codon 59,61)	chr1:115256536, chr1:115256529	TTCGCCTGTCCTCATGTATTGGTCTCTCAT GGCACTGTACTCTTCTTGTCCAGCTGTAT CCAGTATGTCCAACAAACAGGTTTCACC ATCTATAACCACTTGTTTTCTGTAAGAATC CTGGGGGTG
	Exon 4 (codon 117,146)	chr1:115252284 / chr1:115252206	GCACAAATGCTGAAAGCTGTACCATACC TGTCTGGTCTTGGCTGAGGTTTCAATGAA TGGAATCCCGTAACTCTTGGCCAGTTCGT GGGCTTGTTTTGTATCAACTGTCCTTGTTG GCAAATCACACTTGTTTCCCACTAGCACC ATAGGTACATCATCCGAGTCT

BRAF	codon 600	chr7:140453135	CCACAAAATGGATCCAGACAACTGTTCA<br>AACTGATGGGACCCACTCCATCGAGATTT<br>CACTGTAGCTAGACCAAAATCACCTATTT<br>TTACTGTGAGGTCTTCATGAAGAAATATA<br>TCTGAGGTGTAGTAAGTAAAGGAAAACA<br>GTAGATCTCATTTTCCTATCAGAGCAAGC
