# Supplementary material for: Comparison of Next-Generation Sequencing and Polymerase Chain Reaction for Personalized Treatment-Related Genomic Status in Patients with Metastatic Colorectal Cancer
Source: Curr Issues Mol Biol. 2022 Apr 5;44(4):1552–63. doi: 10.3390/cimb44040106 (PMC9164059; doi:10.3390/cimb44040106)
Supplement: Supplementary file 1 [file cimb-44-00106-s001.zip › Supplementary table 1.pdf]

Supplementary Table S1. The primers used in PCR amplification of *KRAS*, *NRAS*, and *BRAF*.

Target genes	Mutational hotspots	Forward primer	Reverse primer
KRAS	Exon 2	5'TCATTATTTTTATTATAAGGCCTG CTGAA -3'	5'- CAAAGACTGGTCCTGCACCAGTA -3′
	Exon 3	5'-TGG AGA AAC CTG TCT CTT GGA-3′	5'-TTT AAA CCC ACC TAT AAT GGT GAA-3′
	Exon 4	5'-TTT TTC TTT CCC AGA GAA CAA AT-3′	5'-AAG AAG CAA TGC CCT CTC-3′
NRAS	Exon 2	5'-GAT GTG GCT CGC CAA TTA AC -3′	5'-GAA TAT GGG TAA AGA TGA TCC GA-3′
	Exon 3	5'- CCC AGG ATT CTT ACA GAA AAC-3′	5'-TAA TAT CCG CAA ATG ACT TGC-3′
	Exon 4	5'-CCC GTT TTT AGG GAG CAG AT-3′	5'-TGC ACA AAT GCT GAA AGC TG-3′
BRAF	codon 600	5'-CATAATGCTTGCTCTGATAGG AAA-3'	5'-TCAGCACATCTCAGGGCCAAA -3'
