# Supplementary figures and images for: Comparison of Next-Generation Sequencing and Polymerase Chain Reaction for Personalized Treatment-Related Genomic Status in Patients with Metastatic Colorectal Cancer
Source: Curr Issues Mol Biol. 2022 Apr 5;44(4):1552–63. doi: 10.3390/cimb44040106 (PMC9164059; doi:10.3390/cimb44040106)

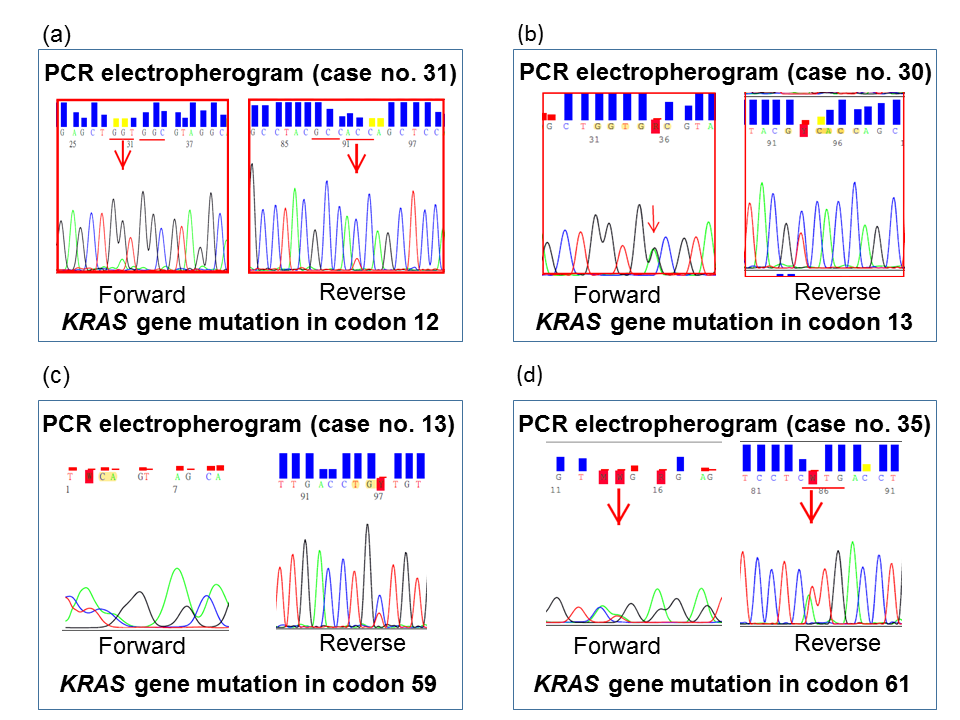

Supplement: Supplementary file 1 [file cimb-44-00106-s001.zip › supple-fig 21 abcd-electropherogram.tif]

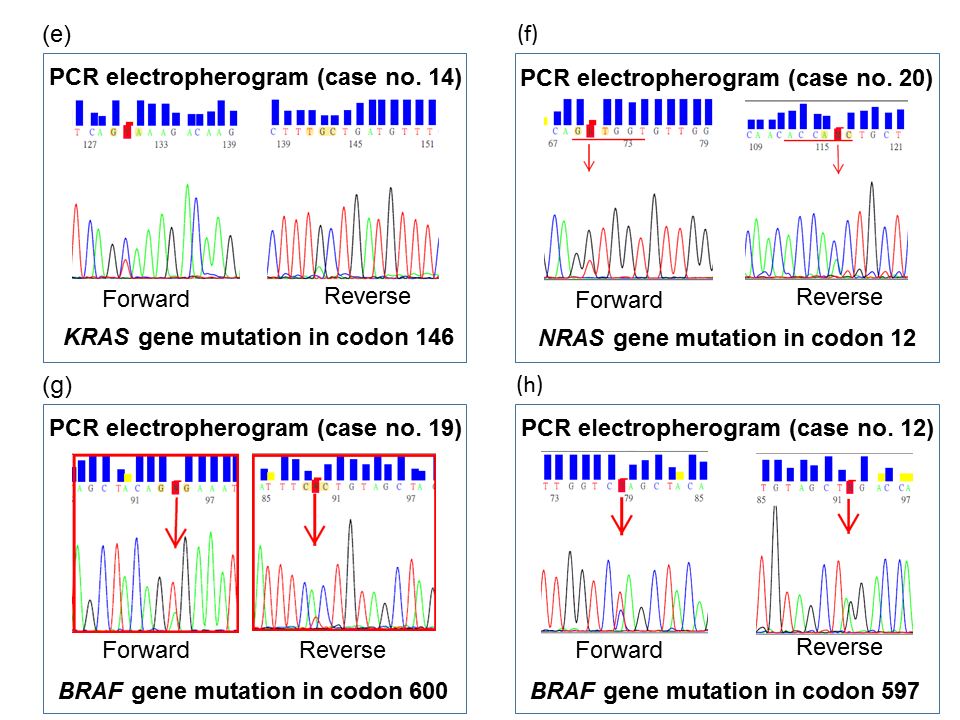

Supplement: Supplementary file 1 [file cimb-44-00106-s001.zip › supple-fig 21 efgh-electropherogram.tif]

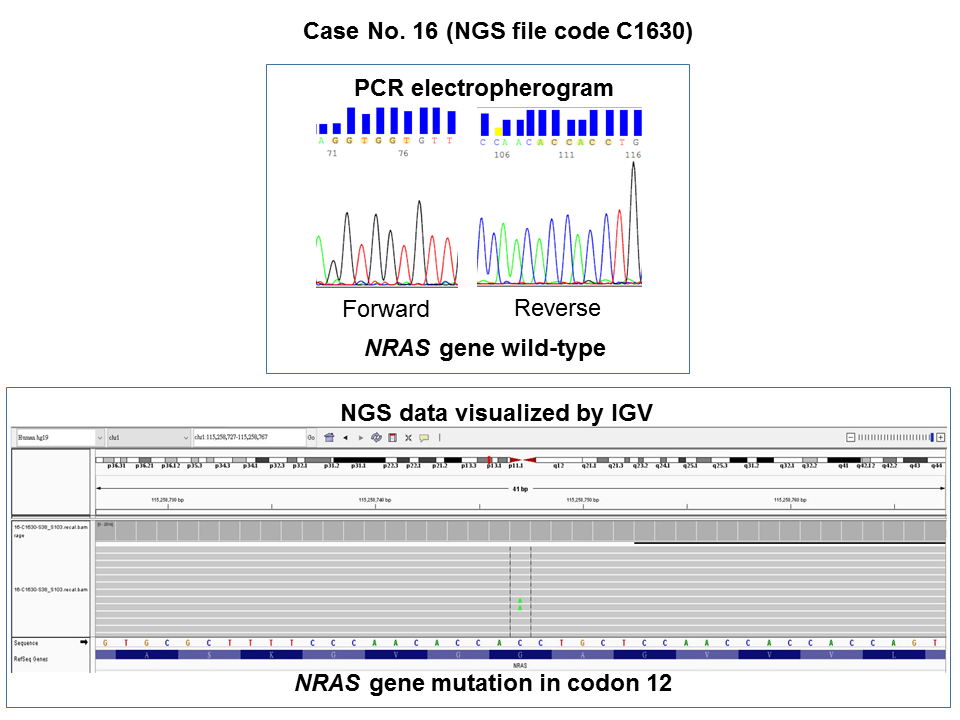

Supplement: Supplementary file 1 [file cimb-44-00106-s001.zip › supple-fig 6-Case No. 16 NRAS.tif]

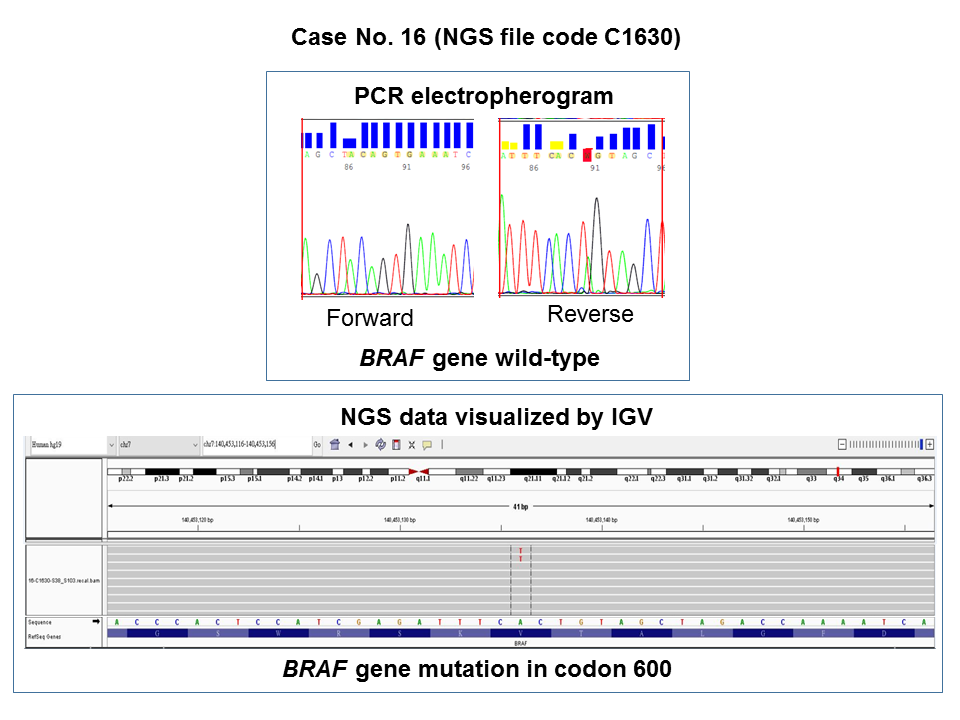

Supplement: Supplementary file 1 [file cimb-44-00106-s001.zip › supple-fig 7-Case No. 16 BRAF.tif]

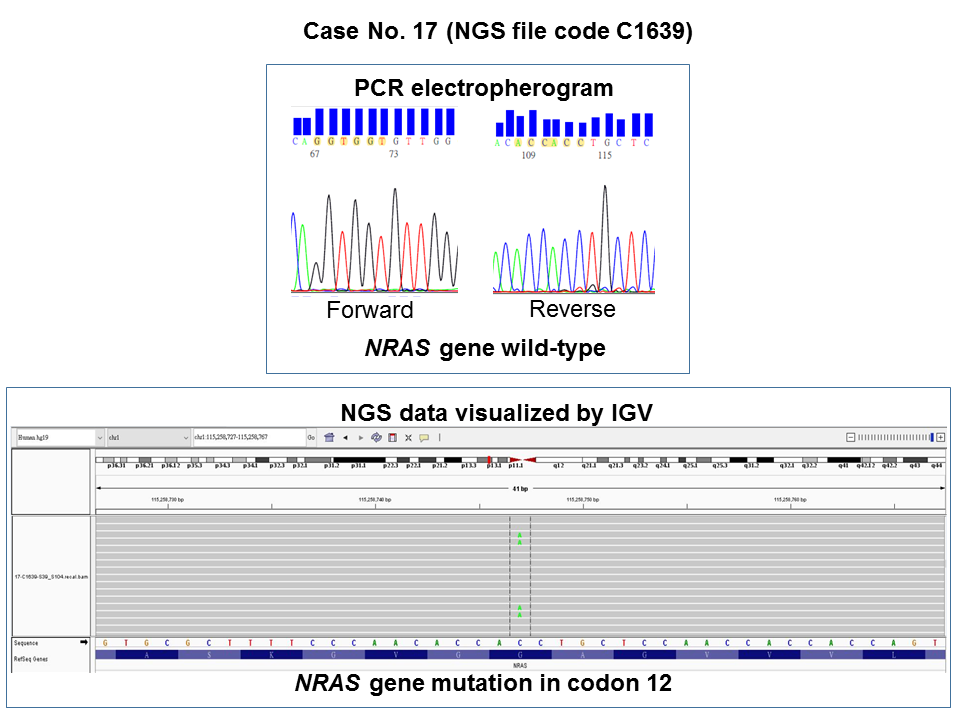

Supplement: Supplementary file 1 [file cimb-44-00106-s001.zip › supple-fig 8-Case No. 17 NRAS.tif]

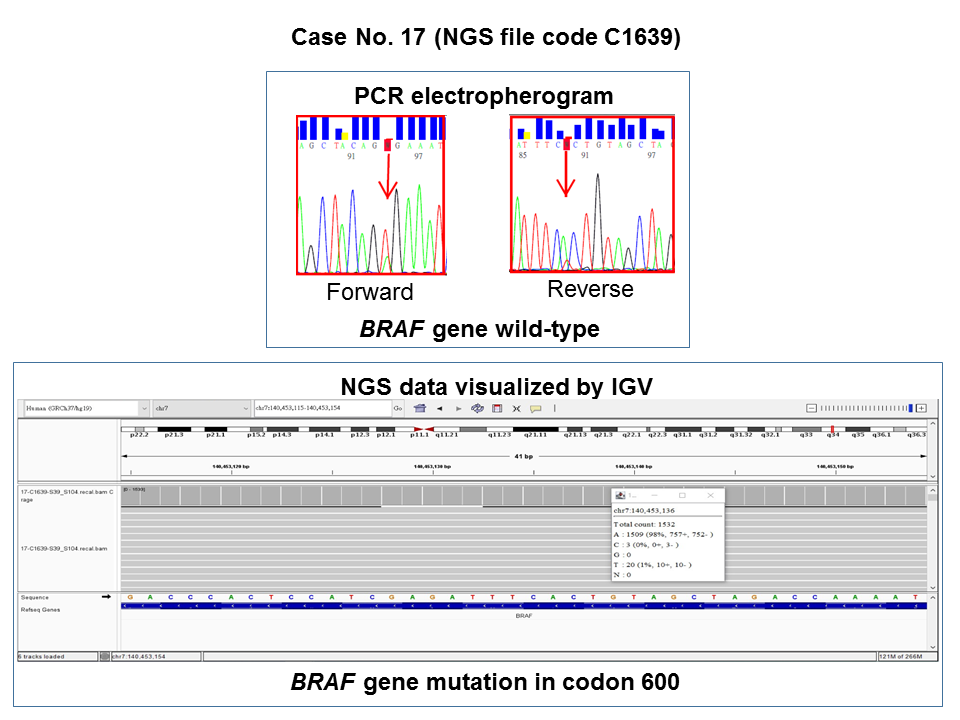

Supplement: Supplementary file 1 [file cimb-44-00106-s001.zip › supple-fig 9-Case No. 17 BRAF.tif]

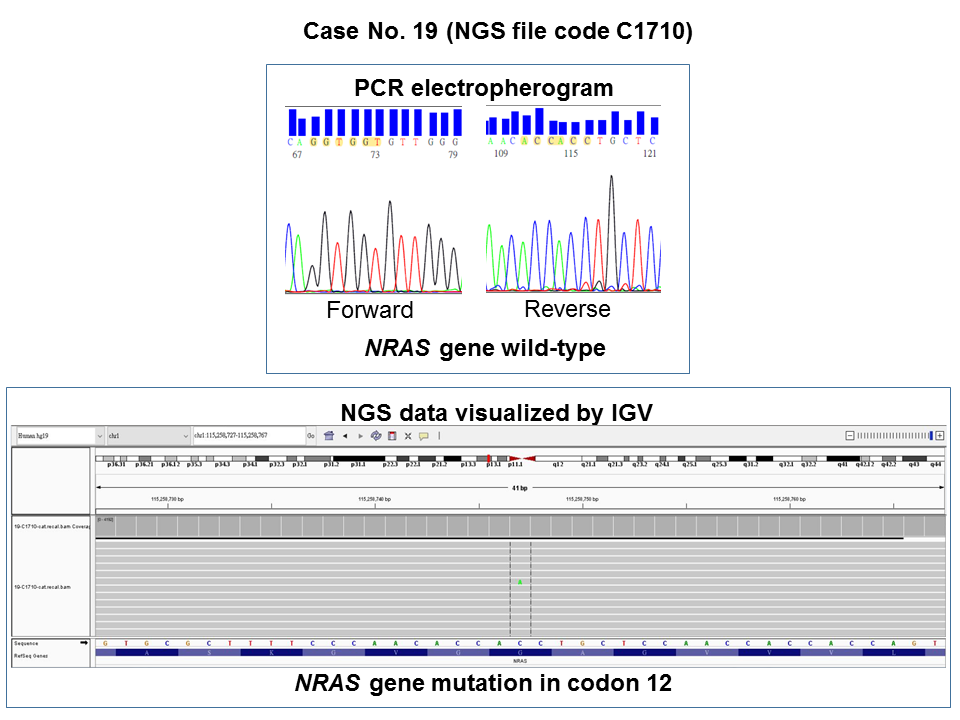

Supplement: Supplementary file 1 [file cimb-44-00106-s001.zip › supple-fig 10-Case No. 19 NRAS.tif]

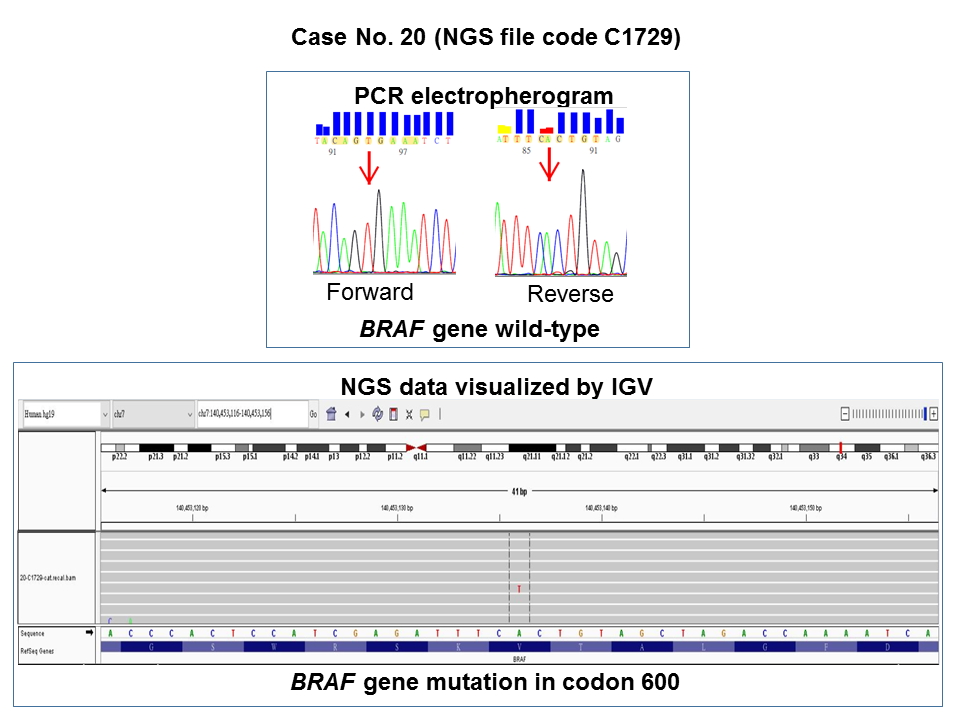

Supplement: Supplementary file 1 [file cimb-44-00106-s001.zip › supple-fig 11-Case No. 20 BRAF.tif]

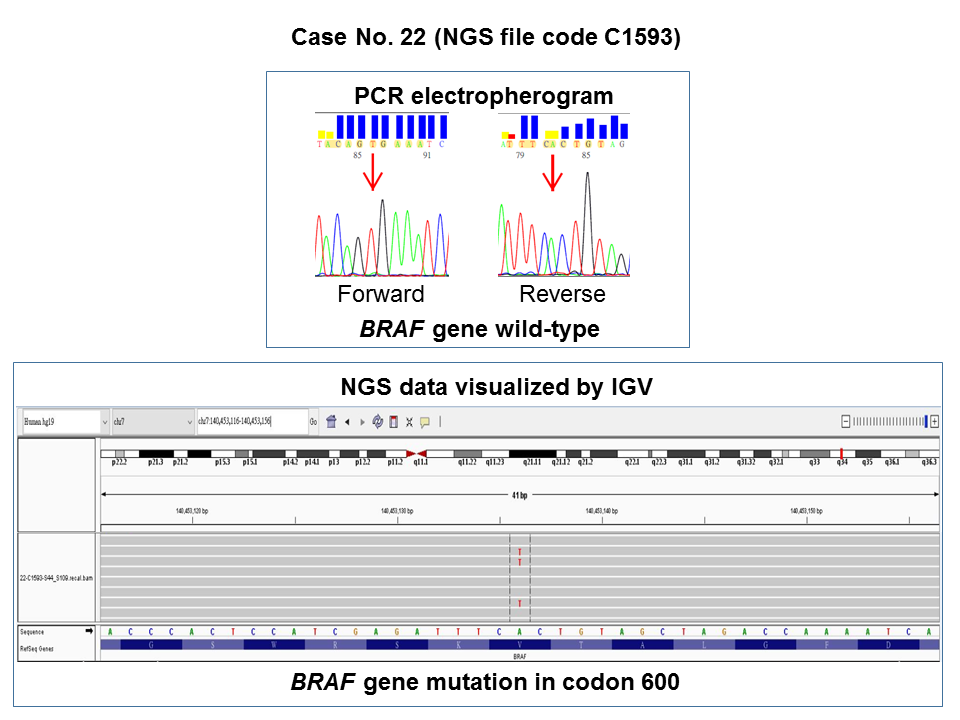

Supplement: Supplementary file 1 [file cimb-44-00106-s001.zip › supple-fig 12-Case No. 22 BRAF.tif]

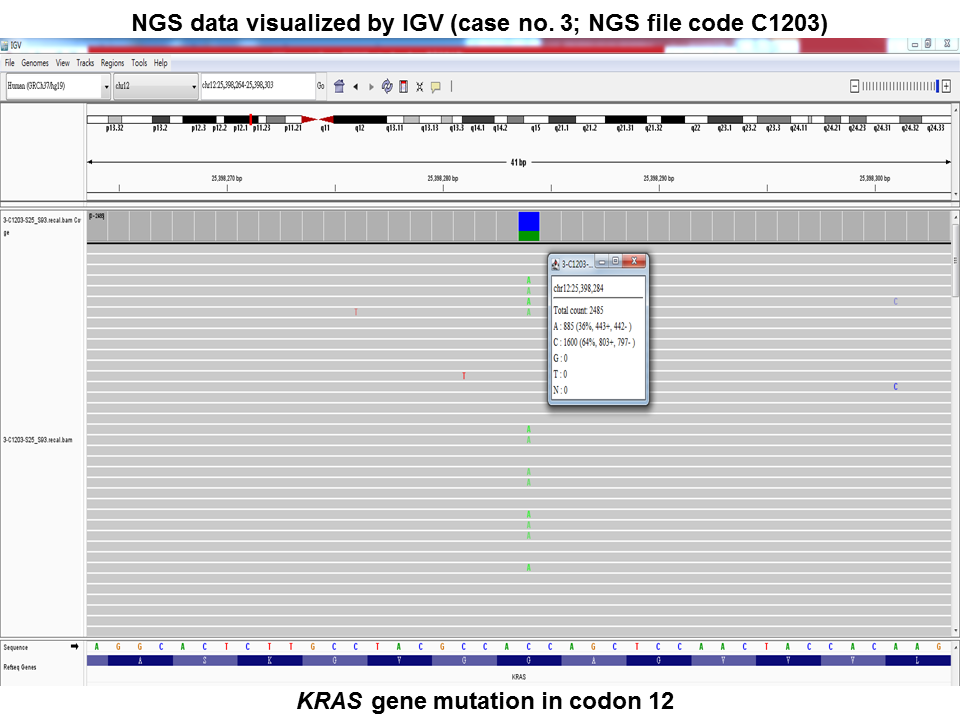

Supplement: Supplementary file 1 [file cimb-44-00106-s001.zip › supple-fig 16-Case No. 3-KRAS codon 12.tif]

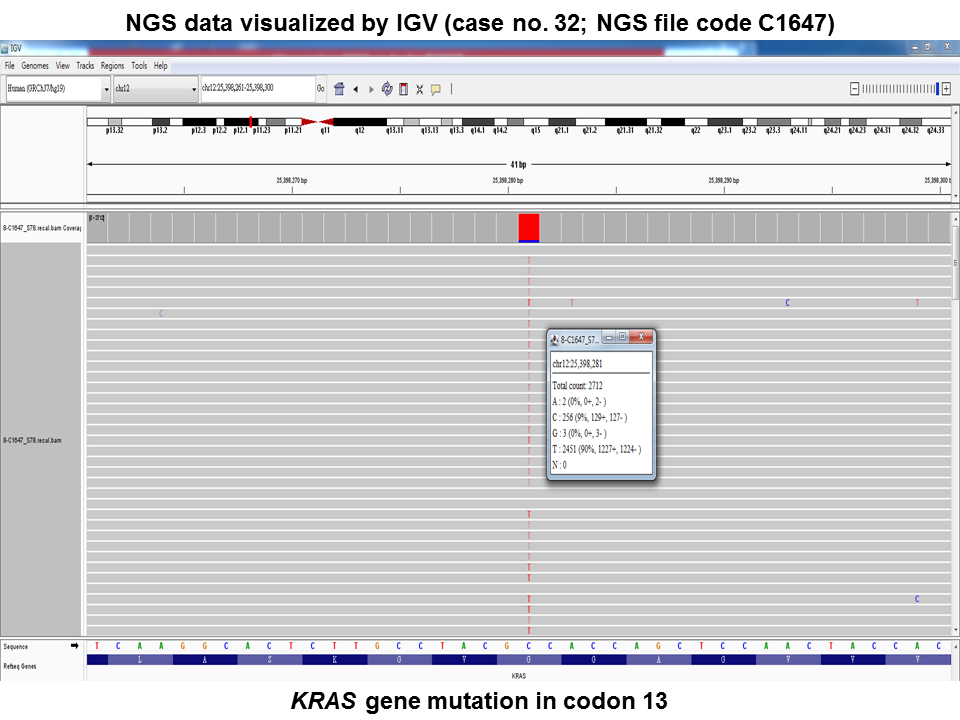

Supplement: Supplementary file 1 [file cimb-44-00106-s001.zip › supple-fig 17-Case No. 32-KRAS codon 13.tif]

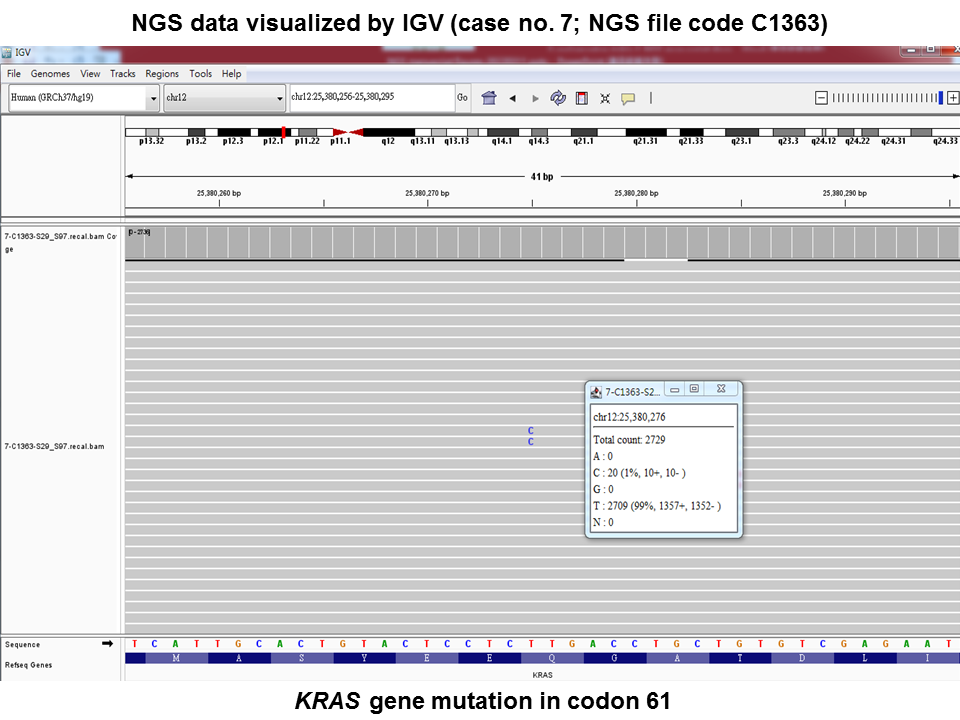

Supplement: Supplementary file 1 [file cimb-44-00106-s001.zip › supple-fig 18-Case No. 7-KRAS codon 61.tif]

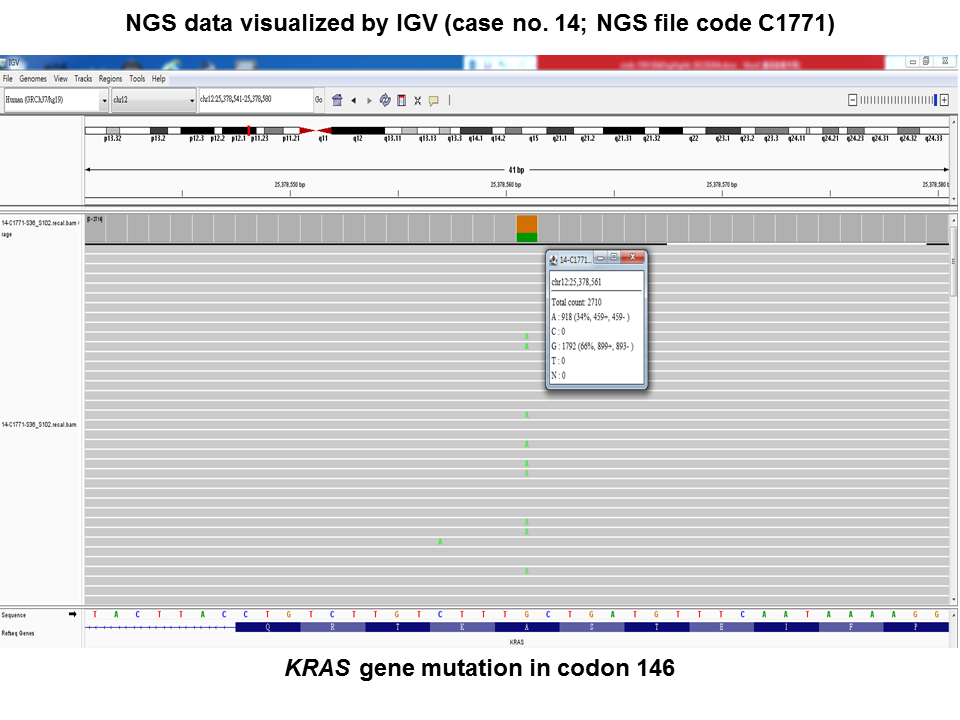

Supplement: Supplementary file 1 [file cimb-44-00106-s001.zip › supple-fig 19-Case No. 14-KRAS codon 146.tif]

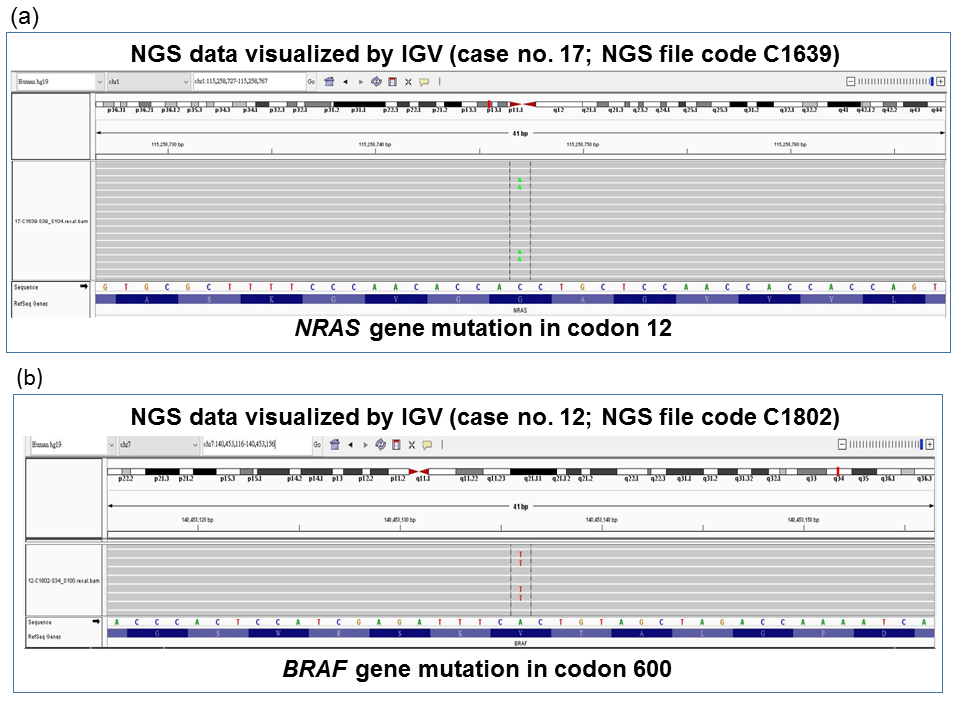

Supplement: Supplementary file 1 [file cimb-44-00106-s001.zip › supple-fig 20-Case No. 17-NRAS codon 12 and case no.12 BRAF codon 600.tif]

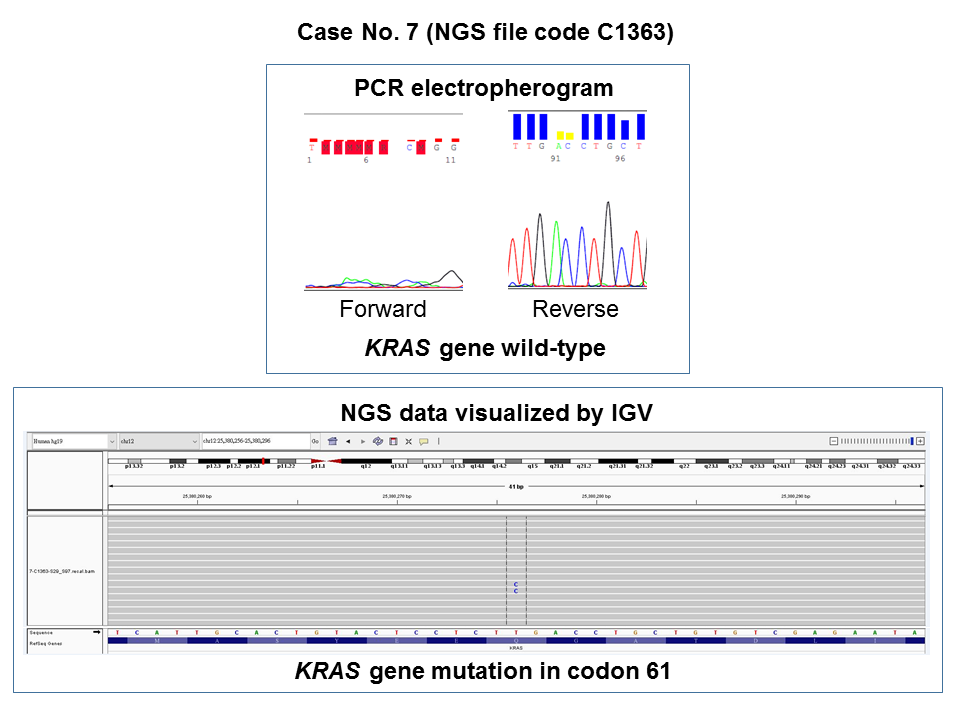

Supplement: Supplementary file 1 [file cimb-44-00106-s001.zip › supple-fig 2-Case No. 7-KRAS.tif]

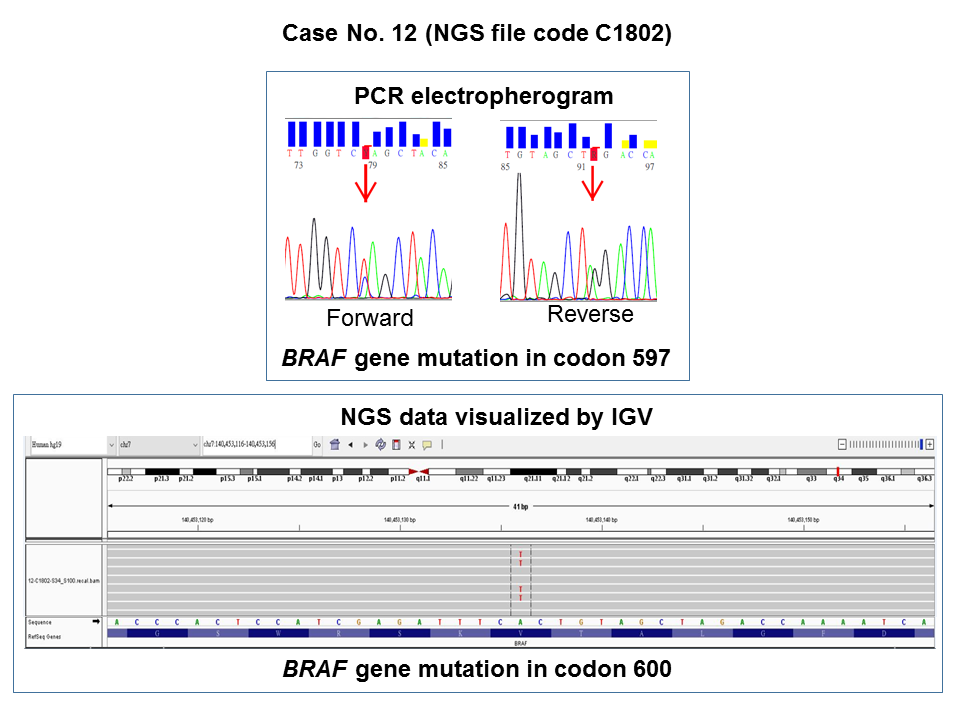

Supplement: Supplementary file 1 [file cimb-44-00106-s001.zip › supple-fig 3-Case No. 12-BRAF.tif]

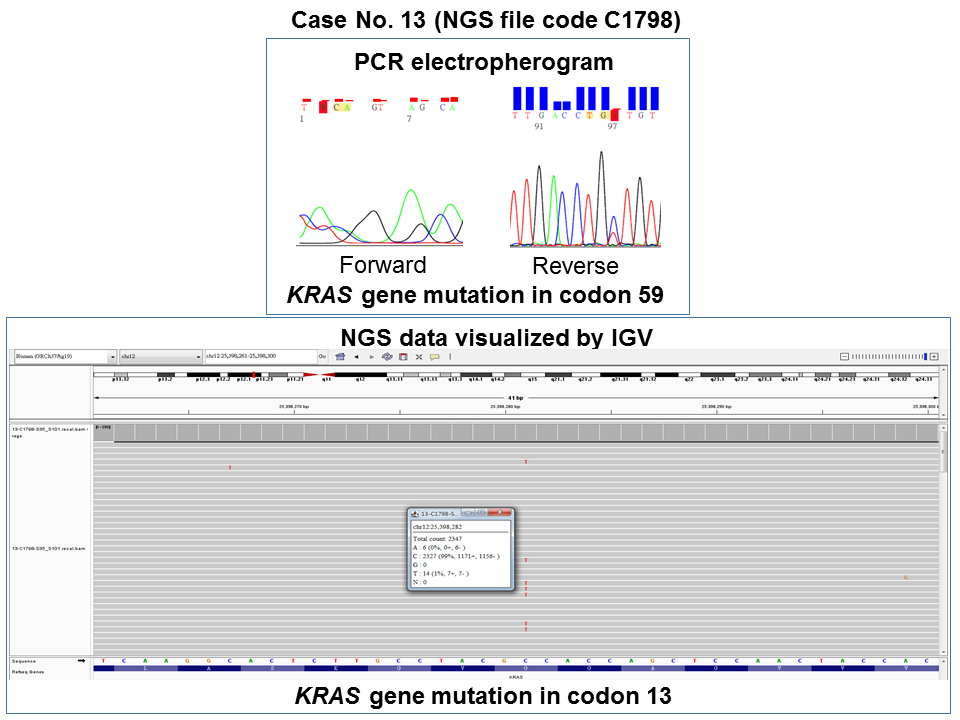

Supplement: Supplementary file 1 [file cimb-44-00106-s001.zip › supple-fig 4-Case No. 13-KRAS.tif]

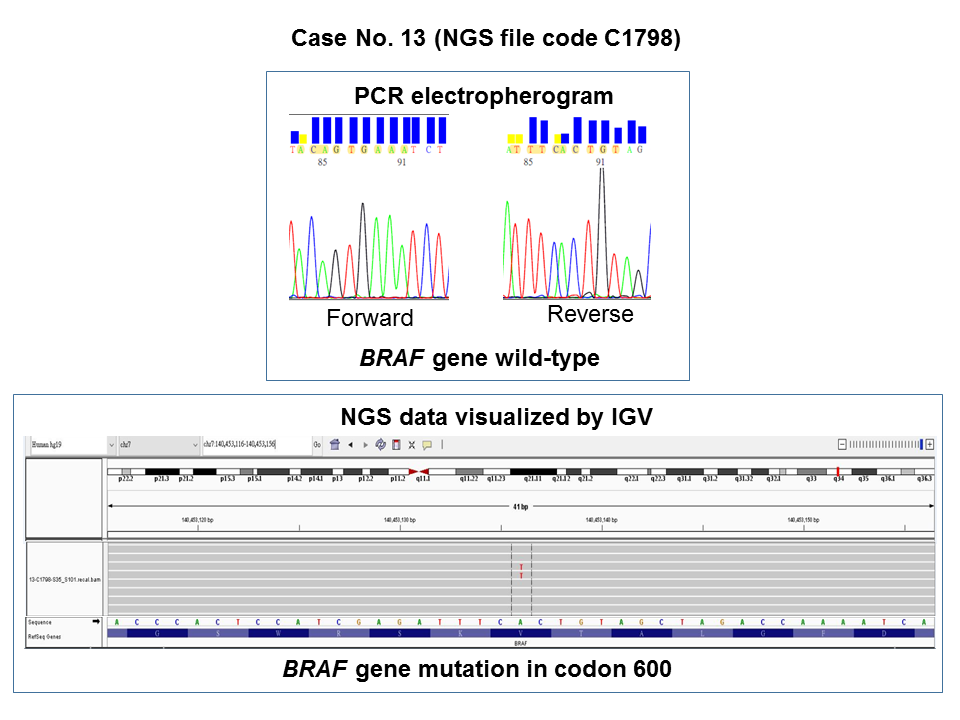

Supplement: Supplementary file 1 [file cimb-44-00106-s001.zip › supple-fig 5-Case No. 13-BRAF.tif]

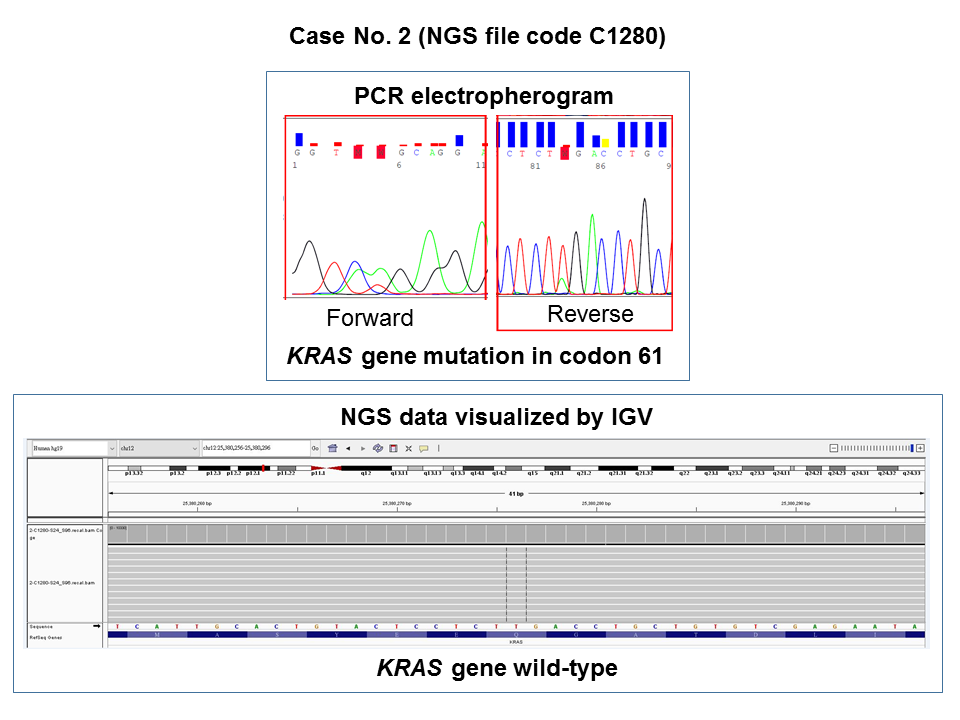

Supplement: Supplementary file 1 [file cimb-44-00106-s001.zip › supple-fig 1-Case No. 2-KRAS.tif]

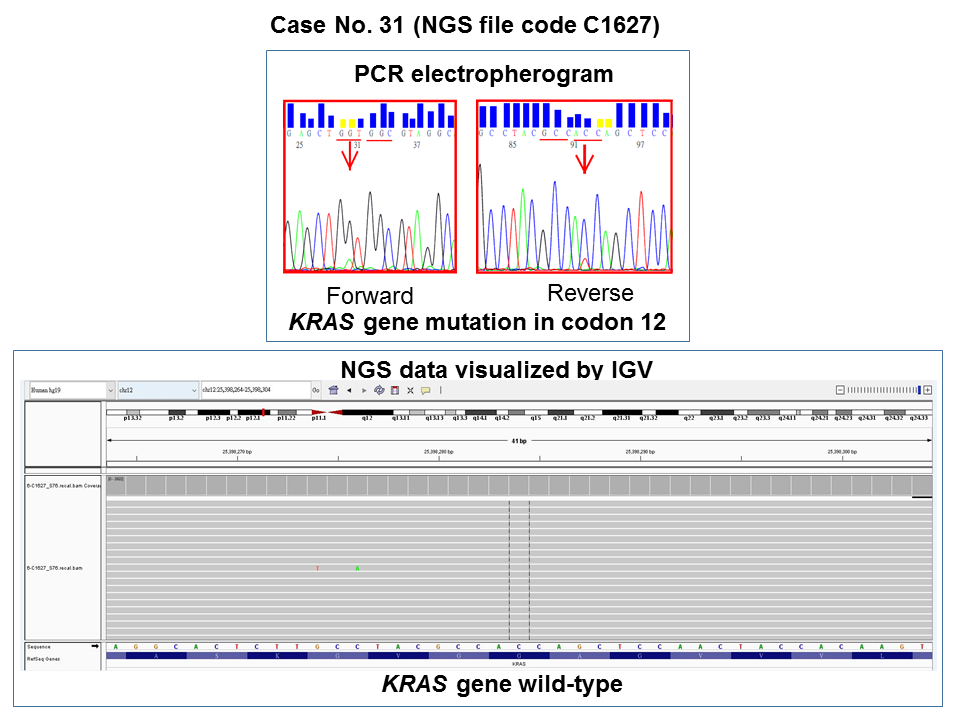

Supplement: Supplementary file 1 [file cimb-44-00106-s001.zip › supple-fig 14-Case No. 31 KRAS.tif]

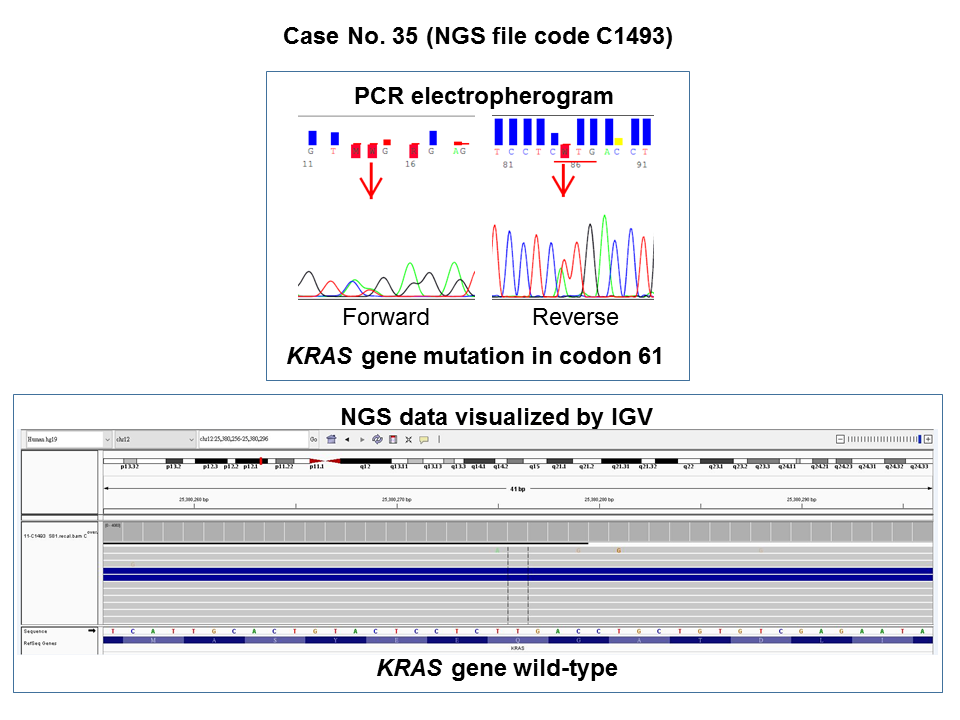

Supplement: Supplementary file 1 [file cimb-44-00106-s001.zip › supple-fig 15-Case No. 35 KRAS.tif]

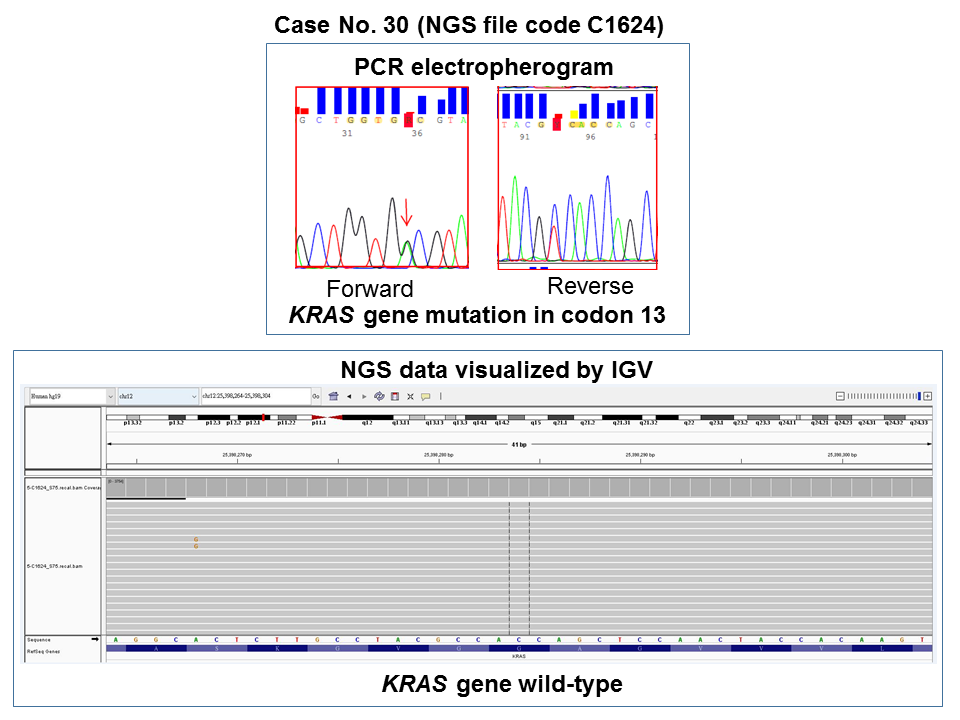

Supplement: Supplementary file 1 [file cimb-44-00106-s001.zip › supple-fig 13-Case No. 30 KRAS.tif]
